# Differential Impact of Science Policy on Subfields of Human Embryonic Stem Cell Research

**DOI:** 10.1371/journal.pone.0086395

**Published:** 2014-04-09

**Authors:** Seongwuk Moon, Seong Beom Cho

**Affiliations:** 1 Graduate School of Management of Technology, Sogang University, Sinsu-Dong, Mapo-Gu, Seoul, Korea; 2 Division of Biomedical Informatics, Center for Genome Science, National Institute of Health, KCDC, Cheongwon-gun, Chungcheongbuk-do, Korea; University of Sao Paulo - USP, Brazil

## Abstract

In this research, we examine how restrictive policy influenced performance in human embryonic stem cell research (hESC) between 1998 and 2008. In previous research, researchers argued whether restrictive policy decreased the performance of stem cell research in some nations, especially in the US. Here, we hypothesize that this policy influenced specific subfields of the hESC research. To investigate the selective policy effects, we categorize hESC research publications into three subfields—derivation, differentiation, and medical application research. Our analysis shows that restrictive policy had different effects on different subfields. In general, the US outperformed in overall hESC research throughout these periods. In the derivation of hESC, however, the US almost lost its competence under restrictive policy. Interestingly, the US scientific community showed prominent resilience in hESC research through international collaboration. We concluded that the US resilience and performance stemmed from the wide breadth of research portfolio of US scientists across the hESC subfields, combined with their strategic efforts to collaborate internationally on derivation research.

## Introduction

Human embryonic stem cell (hESC) research has huge scientific and medical potential but has provoked fierce controversy: the possible destruction of human embryos during the derivation of hESC lines has elicited serious ethical issues from religious and political communities. In 2001, the US president George W. Bush announced that federal funding would not be provided for research on the hESC lines derived after August 2001. While this federal “embargo” on hESC research was effective in the US, many other countries with a more liberal stance toward hESC research continued to invest public funds in this field. Thus, US scientists and policymakers began to debate whether the US performance in hESC research would be damaged by the Bush's restrictions [1]. Rapid progress by such countries as China, Israel, Korea, and Singapore ignited intense debate over whether the US could still sustain scientific performance in this promising research. Although it was subsequently shown to be fraudulent, the announcement by Korean scientist Dr. Hwang Woo-Suk that patient-specific hESC lines had been derived using a somatic cell nuclear transfer method caused a stir among US scientists over the restrictiveness of stem cell policies [2].

Has the restrictive Bush's policy really caused US to lag behind the international competition in hESC research? Scientists have suggested different answers to this question. Owan-Smith and McCormick insist that any lag in productivity of the US in hESC research began after implementation of Bush's policy [1]; Levine also argues that the US has underperformed in published hESC research due to the federal funding restrictions and a shift of scientists into the private sector, where they have less incentive to publish [3]. In contrast, based on the share and quality of the hESC publications by US researchers, Löser *et al.* argue that the US has maintained its performance in hESC research under the Bush administration [4]. They suggest that the different assessments of US performance may be due to different definitions [4]: if hESC research is defined as articles citing the seminal 1998 Thomson paper [3], [5], then US performance would seem to have declined during the Bush administration. If hESC research is defined as articles deriving and using hESC lines, however, US performance would seem to have been maintained [4]. In this research, we investigated the issue in the subfields of hESC research. We chose this method of analysis because the 2001 Bush restrictions prohibited the derivation of additional hESC lines using federal funds, but did not directly restrict non-derivation research. Thus, the restrictions were likely to have different effects across subfields of hESC research, and the evaluation of the US performance in hESC research may differ, accordingly, by subfields. To address issues of whether the trends were due to the restrictive policy or to the other factors, such as globalization of science, we use research articles on RNA interference (RNAi) as our comparison set. This is because RNAi is another breakthrough research area by US scientists but one not likely to be influenced by the Bush policy [3], [6].

## Materials and Methods

### The identification of hESC articles

In order to construct hESC research publication data, we used research articles in journals listed in the Science Citation Index (SCI) database from 1998 to 2008. We chose 1998 as the starting year of our research because it was in that year that James Thomson published his breakthrough article in hESC research. We searched articles using the keyword string “human AND (embryo OR embryonic) AND stem cell*,” which was used by Owan-Smith and McCormick [1]. The use of journal articles in the SCI database excludes data reported from conferences, book series, letters, meeting abstracts, and review materials. This method also excluded articles not in the SCI journals.

Although the SCI database is regarded as highly reliable among researchers, we double-checked to see whether these articles were related to hESCs by using the PubMed database. We examined and confirmed whether the articles from the ISI Science Citation Index database were also listed in the PubMed database and were related to hESCs. We matched the PubMed identifier (PMID) with the ISI identifier using the Batch Citation Matcher (www.ncbi.nlm.nih.gov/pubmed/batchcitmatch/) in the PubMed website and selected the intersection of the articles from the ISI and PubMed databases. Although this data set may not include every single article on hESCs, it is comprehensive in the sense that it was obtained from two important databases in the field of biomedical science.

Finally, we double-checked this list of hESC research articles by asking three hESC researchers in Korea National Institute of Health to confirm whether articles in our list were about hESC research. In order to construct RNAi research publication data as a comparison group, we used articles citing the seminal paper of Fire *et al.* [7] until 2008. Although RNAi research is another scientific breakthrough by the US based researchers, it has been much less politically controversial than hESC research and thus has been used as a comparison group of hESC research in other studies [3], [6].

### The categorization of hESC articles

We categorized these articles into three research subjects: the derivation of hESC lines, the differentiation or development of stem cells into other cell types or organs, and research on the medical application of stem cells. The reason was because the objectives of hESC research range from fundamental understanding of hESC to the use of such knowledge for medical treatment; as Thomson points out in his seminal paper [5], “These cell lines should be useful in human development biology, drug discovery, and transplantation medicine.” The distinction between research on derivation and on the use of hESC lines is also noted by other authors [3], [4], [8]. Because the topics in hESC research mainly include the derivation and differentiation of hESCs that is fundamental to developing future medical applications, we believed that the categorization of hESC research into these three subfields is useful for evaluating the changes of the US performance in a more detailed way.

Regarding categorization, we retrieved the PMIDs of the hESC-related articles and assigned a category to each article using the MeSH and general terms in PubMed. To choose the representative terms for each subfield, we used MeSH terms that were representative to the subfield. Then, to extend the coverage of articles, we chose general keywords similar to the representative MeSH terms. Finally, using the keywords as input search terms, we retrieved the subfield-related articles from Pubmed. Since the keyword-based retrieval collected non-hESC articles, we selected the intersection of the retrieved articles and the hESC-related articles. For retrieving derivation research publications, ‘in vitro fertilization’, nuclear transfer techniques' and ‘clone cells’ were used for key words. For differentiation publications, ‘cell differentiation’, ‘development’ and ‘organ’ were search key words. ‘Biomedical research’, ‘clinical research’, ‘medical research’, ‘application’, ‘treatment’ and ‘therapeutics’ were used for key words of retrieving medical application publications. Each keyword and ‘human’ and ‘embryonic stem cell’ were used for input search terms with ‘and’ Boolean operator in the Pubmed system.

We also confirmed our categorization when we asked three hESC researchers in the Korea National Institute of Health to evaluate whether our categorization was sensible. In our data, one article could be related to more than one subfield. For instance, an article could be recorded as both derivation and differentiation research.

### Identification of nationality and international collaboration

We determined the nationality of each article based on the address of the authors' institutions. For instance, if the author of a paper worked for a university in a country, we recorded that the article as one belonging to that country. An article with multiple institutional affiliations was recorded in each of the nationality categories. Thus, for instance, an article could be regarded as from both the Korea and US. The hESC articles were from 28 countries: Australia, Austria, Belgium, Canada, China, Czech Republic, Denmark, Finland, France, Germany, Greece, Hungary, India, Iran, Israel, Italy, Japan, Korea, the Netherlands, Russia, Singapore, Spain, Sweden, Switzerland, Taiwan, Turkey, the UK, and the USA. The RNAi articles were from 43 countries including the 28 countries in the hESC research data. Articles with multiple nationalities were considered to be the result of international collaboration.

### Measure of research opportunity

We used country's policy attitude toward the derivation and use of the hESC lines as a measure of research opportunities. The Hinxton Group (http://www.hinxtongroup.org) categorized countries' stem cell policies into four types—permissive, permissive compromise, restrictive compromise, and prohibitive—based on how much restriction was put on the derivation and use of hESC lines through in vitro fertilization (IVF), and somatic cell nuclear transfer (SCNT) for research purpose. The criteria for this categorization are listed in Table S1.

We assigned numbers to each type: 1 to permissive, 2 to permissive compromise, 3 to restrictive compromise, and 4 to prohibitive. We calculated the mean and standard deviation of the “permissiveness score” for all participating countries per article. Because the permissiveness score of the US was 2, articles with the score lower than 2 imply that US scientists collaborated with scientists in countries with more permissive environments. The standard deviation would be zero if collaborating countries had the same policy type; it would become larger if collaborating countries had different policy types. For instance, if all collaborating countries adopted the permissive policy, the standard deviation turned out to be zero; if one country adopted a permissive policy and the other, collaborating one adopted a restrictive compromise policy, then the standard deviation would be 0.25.

### Binomial probability test of whether the US was lagging in derivation research

Using the exact binomial probability, we tested whether each country contributed to a specific subfield more (or less) as compared to its contribution to overall hESC research. Here, we used the percentage of all hESC research publications by each country as the assumed probability of the country's publishing in each specific subfield, and then tested whether the country published in each subfield with the same assumed probability. Our null hypothesis was that the contribution of a country to each subfield was the same as its contribution to overall hESC research. Our alternative hypotheses were one-sided: if the null hypothesis was rejected in a certain subfield, we interpreted the rejection as showing that the country contributed to the subfield either more than or less than overall hESC research. The *p*-value in the following tables represents the lower one-side *p*-value when an observed number is less than the expected one, and the upper one-sided *p* value when an observed number is larger than the expected one. Table S2 represents the results from all countries in the whole period between 1998 and 2008. Table S3 shows results from 13 countries that contributed in all three of the sub-periods (1998–2001, 2002–2005, 2006–2008).

## Results and Discussion

### Identification of hESC publications

In this research, we identified 993 unique hESC research articles, published in 230 journals; the authors were affiliated with 1035 institutions (Table S7). Our data showed that the geography of hESC research has been globally diffused. In terms of the number of institutions affiliated with researchers, the top three countries among a total of 28 are the US, the UK, and China; 282 institutions are based in the US (27.25%), 104 institutions in the UK (10.05%), and 62 institutions in China (5.99%). Our RNAi comparison set was constructed by using all articles citing the seminal Fire and Mello's paper [3], [7]. These procedures generated 2,553 unique RNAi research articles, published in 574 journals (Table S8).

In the results of the categorization analysis of the hESC publications, the most active research topic was the differentiation process of hESC (Table S7): 618 articles (62.2%) are reported as differentiation research. This topic generated an increasing number of reports even after 2001 when Bush's restrictive policy was addressed; the portion of articles reporting differentiation research increased from 21.42% in 1998 to 63.54% in 2008. This trend, as of 2008, implies that the international scientific community recognized that the mechanism of stem cell differentiation was an urgent research topic. The possible medical application of hESCs was the second-most active research area (180 articles, or 18.2%). Despite interest from the public media and policymakers, only 94 articles (9.5%) were related to the derivation and maintenance of hESC lines.

### International distribution of hESC research

**Figure 1:**
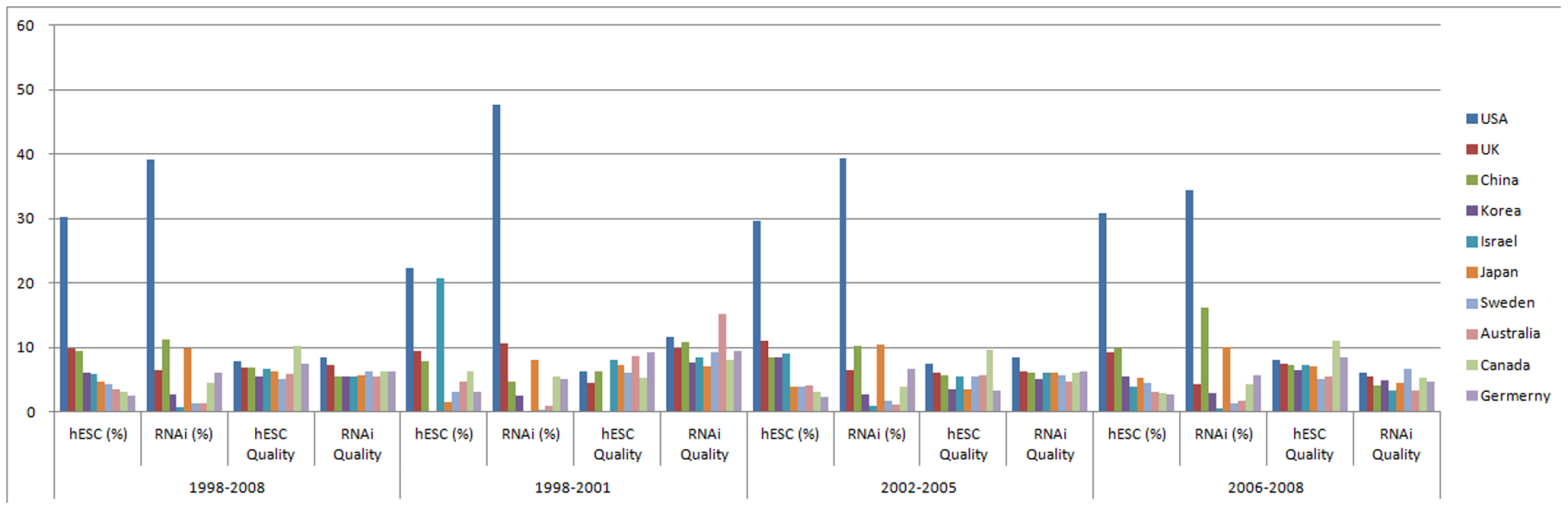
Contribution to hESC Research. This figure contains top 10 countries in terms of percentage in hESC research. Y axis indicates percentage (%).

Figure 1 shows that the US did not lag behind in stem cell research between 1998 and 2008 (See Table S4 for details). Rather, the US was advancing ahead of all the other countries in hESC research. During this period, the share of US publications in overall hESC research was 30.15%, the highest among all countries. Moreover, the US was far ahead of every other country. For instance, the publications from the UK, the second-most active country in hESC research, were just 9.76% of the total. Despite the restrictions in place since 2001, the share of US publications actually grew: from 22.22% in the period 1998–2001 to 29.67% and 30.84% in the period 2002–2005 and 2006–2008, respectively. The opposite pattern held in RNAi research: the share of US publications in RNAi research continued to decline from 47.44% in the period 1998–2001, to 39.38% and 34.37% in the period 2002–2005 and 2006–2008, respectively. Because research on RNAi was not systematically discouraged like hESC research during these periods, it seemed to demonstrate the evolution of a less contentious, breakthrough scientific field [3], [6]. Since these important fields show opposite trends, the idea that the US has basically maintained its performance in hESC research seems to be supported.

The average journal impact factor, provided by Thomson Reuters, for US articles published between 1998 and 2008 was 7.87. Using this measure, the US was ranked fifth among 28 countries. When evaluated with different periods, the impact factor of US publications improved from 6.31 in the period 1998–2001 to 7.45 in 2002–2005 and 8.10 in 2006–2008, respectively. The impact factor of US publications in RNAi research, however, declined. Although the impact factor of US publications was the highest among all countries by a large amount in period 1998–2001, the measure declined to levels similar to other countries in subsequent periods. So, these results indicated that US scientists' contribution to overall hESC research increased during this era.

### Research activities on subfields of hESC research

If that was the case, why was the US scientific community concerned that its advance in hESC research was being delayed by the Bush administration? Did the US scientific community overreact to the Bush policy, or did that policy have a genuinely negative impact on US performance in hESC research? If the Bush policy had a harmful effect on US performance, how could the US maintain its leading position?

**Figure 2:**
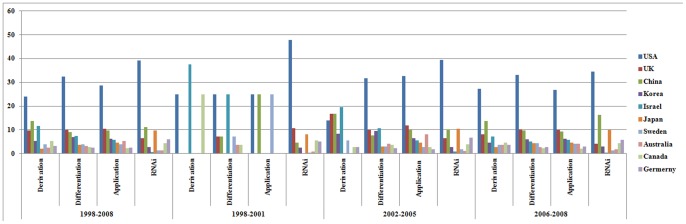
Percentage in Three hESC Research Subfields. As Figure 1, top 10 countries were included in terms of percentage in hESC research. Y axis indicates percentage (%).

The examination of research activities in the three hESC subfields shows that the Bush policy selectively influenced the US performance across the subfields; the policy seemed to delay US advance in derivation research, but not to influence US performance in non-derivation research such as differentiation. Moreover, the examination of three subfields shows that differentiation research accounted for most of the US advance in hESC research under the Bush administration. As can be seen in Figure 2, the US share of derivation research was 24.03%, which was 6.12 percentage points lower than the US share of overall hESC research. On the contrary, the US portion of differentiation research was 32.30%, which was 2.15 percentage points higher than the US portion of overall hESC research (See Table S5 for details). The binomial tests of proportions across hESC subfields show that US scientists contributed to derivation research significantly less (*p* value: 0.05) and to the differentiation research more (*p* value: 0.09) than they did to the overall hESC research (Table S3).

The negative effect of the restrictive policy on hESC derivation and the significance of hESC differentiation are conspicuous in time series patterns (Table S3). Although the US share of derivation research was 25% in the period between 1998 and 2001, the number decreased to 13.89% in the period between 2002 and 2005. However, the US share in differentiation and medical application research actually increased during the same periods (from 25 to 31.62% and from 25 to 32.73%, respectively). Especially, the US share in differentiation research continued increasing to 33.02% in period 2006–2008.

In contrast, countries regarded as catching up to the US in hESC research—for instance, China, Israel, and the UK—focused on derivation research. For instance, during the period between 1998 and 2008, the US generated 24.03% of derivation research publications, compared to 30.15% of overall hESC research publications. During the same period, China generated 13.64% of derivation research publications, although its share of overall hESC research articles was 9.41%; Israel published 11.69% of derivation research, while its portion of overall hESC research publication was 5.92%.

Interestingly, the Germany having the restrictive policy showed similar derivation research performance compared with that of the Sweden having the permissive policy between 1998 and 2008. This finding resulted from the fact that the derivation researches of the Germany were performed via international collaboration.

The increase in derivation research by competing countries during the period between 2002 and 2005—right after the Bush announcement—is also impressive: China increased its share of derivation research by 16.67 percentage points; Korea, by 8.33 percentage points; the UK, by 16.67 percentage points. Although Israel's contribution to publications in this field decreased from 37.5 to 19.44%, Israel still outperformed in derivation research. Considering the US portion of derivation research in this period (13.89%), the concern of the US scientific community that the Bush's policy was retarding US progress seemed to originate from the fact that the US was lagging behind in derivation research—the subfield that the 2001 restriction afflicted directly. The binomial test confirmed that during the period between 2002 and 2005 (as noted above, this was immediately following Bush's announcement), the US contribution to derivation research decreased significantly, compared to its contribution in that period to overall hESC research (*p* value 0.02, Table S3). During that same time, China and Israel contributed to derivation research more than to overall hESC research (*p* values are 0.08 and 0.04, respectively).

Thus, the concern that the US was lagging behind in hESC research seemed to reflect this relative US decline in *derivation research*, despite the fact that the US was actually leading in overall hESC research and, especially, in differentiation research. In contrast to their rapid growth in derivation research, however, the countries noted above (China, Korea, the UK, and Israel) did not increase their share of differentiation research remarkably. For instance, during the 1998–2001 and 2002–2005 periods, China and the UK increased their contributions to differentiation research only by 0.58 and 2.79 percentage points, respectively. The US increased its contribution to differentiation research by 6.62 percentage points—to 31.62%. These observations indicate that the US was maintaining its performance in hESC research because US scientists actively conducted differentiation and medical application research; the competing countries are catching up with the US only in derivation research.

We performed the same subfield analysis with grouping 7 permissive countries (i.e. UK, China, Korea, Israel, Japan, Sweden and Australia) into one “permissive countries”. We found that the results showed the same tendency with the original subfield analysis. The permissive countries occupied higher share in derivation research by 5.2 percentage points compared with its overall share in hESC research (see Table S6). Moreover, from 2002 to 2005, the period after Bush's restrictive policy was announced, the difference was much larger. The permissive countries' share in the derivation research was 17.8 percentage points larger than their share in overall hESC research, while USA's share was 15.8 percentage points lower than its overall hESC research share. Germany that has the most restrictive policy did not show much change in the share of derivation research.

These publication patterns suggest that scientists' concern about a US lag was grounded in the declining US contribution to derivation research. The careful examination of hESC subfields also suggests a source of US advance in hESC research: the US scientific community had the capability to conduct broader research in the hESC research than did scientific communities in other countries. US scientists were advancing in differentiation and medical application research when they were discouraged from conducting derivation research by the Bush administration.

It might be possible that derivation research of stem cells in the US was less active because enough cell lines became available in the observation period. However, Scott *et al.* [10] reported that only 21 cell lines were actually qualified for stem cell research among the 78 cell lines that were approved by NIH during Bush administration. Moreover, only 4 cell lines were actively used for stem cell researches during the period. These indicated that even hESC lines were not enough for stem cell research of differentiation and medical application, the derivation research was lagged in the US.

### Resilience by international collaboration

Even in derivation research, the US scientists showed resilience after 2005. As Figure 2 shows, after dropping from 23% in the period 1998–2001 to 13.89% in the period 2002–2005, the US share of derivation research rebounded to 27.27% in the period 2006–2008. In contrast, the share of competing countries in derivation research decreased in the 2006–2008 period. For instance, the shares of China, Korea and the UK in derivation research dropped by 3.03, 3.78 and 8.49 percentage points in the period 2006–2008, respectively (Table S5).

One possible source of US resilience in derivation research was international collaboration. If other things are equal, scientists prefer domestic collaboration to international collaboration because of higher transaction costs involved in international collaboration. Even when scientists need to collaborate internationally, they prefer to work with colleagues in countries that have comparable or better research environments than their own. Therefore, US scientists would in general have weak incentives to collaborate internationally in hESC research. The Bush policy that directly prohibited the derivation of hESC lines with federal funds may have influenced the initiation of international collaboration differently across subfields. US scientists may have used international collaboration as a way of continuing their derivation research; however, they had less incentive to collaborate internationally on non-derivation research.

As shown in Table 1, the collaboration patterns in RNAi and the hESC research confirm these conjectures: US researchers did not strongly prefer international collaboration in RNAi research—an important research field in which environments were not restrictive, compared with hESC research—but were more likely to collaborate internationally in hESC research. The percentage of US publications with international collaboration in RNAi research was 22.63% while the percentage in hESC research was 41.37%.

**Table 1:** International Collaboration by US Scientists.

Period	IC	hESC Total	hESC Derivation	hESC Differentiation	hESC Application	RNAi
**1998–2008**	**IC (A)**	175	26	101	39	267
	**US only (B)**	248	11	170	62	913
	**Total (C)**	423	37	271	101	1180
	% of IC (A/C*100).	**41.37**	**70.27**	**37.27**	**38.61**	**22.63**
**1998–2001**	**IC(A)**	10	2	4	1	58
	**US only (B)**	4	0	3	4	221
	**Total (C)**	14	2	7	5	279
	% of IC (A/C*100).	**71.43**	**100.00**	**57.14**	**100.00**	**20.79**
**2002–2005**	**IC (A)**	43	4	35	13	129
	**US only (B)**	65	1	51	23	407
	**Total (C)**	108	5	86	36	536
	% of IC (A/C*100).	**39.81**	**80.00**	**40.70**	**36.11**	**24.07**
**2006–2008**	**IC (A)**	122	20	62	25	80
	**US only (B)**	179	10	116	39	285
	**Total (C)**	301	30	178	64	365
	% of IC (A/C*100).	**40.53**	**66.67**	**34.83**	**39.06**	**21.92**

IC: international collaboration.

The collaboration patterns across the hESC subfields also support these conjectures: US researchers had a strong tendency to collaborate internationally in derivation research while they did not prefer international collaboration for differentiation and medical application research. The percentage of US publications with international collaboration in derivation research was 70.27% during the 1998–2008 period. In differentiation and medical application research, however, such percentages were 37.27 and 38.61, respectively, during the same period. Considering the fact that the Bush policy specifically held up the generation of new hESC cell lines in the US, we conjecture that the restrictive environment induced US hESC derivation researchers to collaborate with scientists in other countries more actively than differentiation and medical application researchers. Thus, despite possible transaction costs, US scientists conducting derivation research *did* strategically use international collaboration to deal with US restrictions.

To examine the existence of such strategic international collaboration, we also measured national differences in research opportunities, based on how much more permissive a collaborating country's legal environment was than the US. We scored each country's permissiveness in hESC research and scaled it from 1 to 4: 1 is “permissive” and 4 is “prohibitive” (Table S1). We examined the mean and standard deviation of the “permissiveness score” for all participating countries for each article. On this scale the lower the number the more permissive the country. The US was assigned a score of 2, which meant “permissive compromise.”

If the mean score of an article was less than 2, this implies that the US scientists collaborated with scientists in countries that were more permissive than the US. As shown in Table 2, when all articles were considered, derivation research showed a greater “permissiveness difference” in the period from 1998 to 2008, than did differentiation or medical application research. The mean score was 1.80, with a standard deviation of 0.58, which was the largest standard deviation. Differentiation and medical application studies showed means of 1.88 and 1.89 with standard deviations of 0.55 and 0.48, respectively. Thus, US scientists who conducted derivation research clearly chose collaborating partners with more permissive legal environments than the US. Those who conducted non-derivation research, however, chose collaborating partners with relatively similar legal environments. In the less contentious field of RNAi research, the average score was 1.93, which suggests that US scientists collaborated with scientists in countries very close to the US in terms of policy permissiveness.

**Table 2:** Policy Similarity between the US and Collaborating Countries.

Period	Derivation (n = 37)	Differentiation (n = 271)	Application (n = 101)	RNAi (n = 1180)
**1998–2008**	1.80 (0.58)	1.88 (0.55)	1.89 (0.48)	1.93 (0.50)
**1998–2001**	1.50 (0.71)	1.76 (0.50)	1.33 (0.58)	1.95 (0.55)
**2002–2005**	1.70 (0.74)	1.88 (0.49)	1.89 (0.50)	1.93 (0.48)
**2006–2008**	1.84 (0.53)	1.88 (0.59)	1.89 (0.47)	1.93 (0.50)

The degree of permissiveness of the United State is 2. The number lower than 2 means “more permissive than the US”. Parenthetical figures represent standard deviation.

Temporal analysis of the policy similarity among collaborating countries also supports the idea that US derivation researchers used international collaboration to exploit their partners' more permissive environments. The standard deviations of permissiveness score in US derivation publication were 0.71 and 0.74 in the period 1998–2001 and 2002–2005, respectively, which were higher than standard deviations from the differentiation in the same periods (0.50 and 0.49, respectively). When the domestic research environment improved after 2005, however, US derivation researchers collaborated with scientists in countries with more similar research environments. The standard deviation reduced from 0.74 in the period 2002–2005 to 0.53 in the period 2006–2008, which was similar to the standard deviation from non-derivation research and RNAi research. The temporal patterns of collaborating partners' legal characteristics in non-derivation and RNAi research did not show much change during these periods. Especially, the stable mean in RNAi research collaboration during these periods implies that the policy permissiveness did not influence the collaboration pattern of US scientists in this less contentious research area (Table 2).

The turnaround of US research environments came through the availability of alternative funding sources and the introduction of more permissive hESC policies by the state governments [6]. Although competing countries began catching up to the US only in derivation research after the 2001 Bush's policy, concern over losses in performance caused the US scientific community to react and become inventive. For instance, local governments and research universities supported by private philanthropy began to give preferential treatment to stem cell research not supported by the federal government [6], [9]. These alternative initiatives may have enabled US scientists to maintain their contribution to hESC research and lessen the need for international collaboration in derivation research.

Taken together, it seemed that the examination of the three subfields of hESC research allows us: (1) to clarify issues of US performance decline and (2) to identify the sources of advance and resilience in the US scientific community. First, the US contributed to the hESC research—especially in differentiation research—more than any other country, even under the Bush administration. The Bush policy, however, delayed advances in derivation research by US scientists. These observations answer the question of whether the US has performance in hESC research. Second, despite the negative effect of the restrictive Bush policy, the US scientific community was resilient. The US scientific community was managing broader research portfolios in hESC research and, in particular, advancing in differentiation hESC research, far ahead of researchers in other countries. US scientists strategically used international collaboration as a way to continue their derivation research right after the Bush announcement. Later in the Bush administration, the availability of diverse funding sources and more permissive state government policies enabled US scientists to resume the lead in research, even in derivation research. These findings also suggest that any evaluation of the effects of a new science policy needs to take into account the responses of the scientific communities that the policy influences.

## Conclusion

We have observed that the US had been leading the hESC research since 1998 and that the Bush policy had differential effects across the subfields of hESC research; US scientists have been productive in the research of hESC differentiation regardless of the restrictive policy, which have been a source of US research performance under the Bush administration. Although US scientists were discouraged from doing derivation research after the Bush announcement, their research on derivation rebounded after 2005. We note that international collaboration between the US and other countries had different patterns across the hESC research subfields under the Bush administration; the international collaboration of US scientists was more active in derivation research than in differentiation and medical application research. Moreover, US scientists doing derivation research collaborated with scientists in countries where the legal stance toward hESC was more permissive than in the US. But collaborating partners in non-derivation research had similar legal stance on hESC research. Therefore, we believe that careful examination of the hESC research subfields can provide an answer to the current controversy over US performance in this important field and can suggest possible bases of US resilience in hESC research under the Bush administration. Our approach may also uncover international players' strong and weak points in hESC research.

## Supporting Information

Table S1 **Categorization of stem cell policies by the Hinxton Group.**
(XLS)

Table S2 **Results of binomial test for contribution of each country in a specific subfield of hESC research between from 1998 to 2008.**
(XLS)

Table S3 **Results of binomial test for contribution of each country in a specific subfield of human embryonic stem cell research in sub-periods (1998–2001, 2002–2005, 2006–2008).**
(XLS)

Table S4 **Results of all hESC publications in each country between 1998 and 2008.**
(XLS)

Table S5 **Results of the sub-field hESC publications in each country between 1998 and 2008.**
(XLS)

Table S6 **Results of the sub-field hESC publications between permissive countries and the others.**
(XLS)

Table S7 **hESC research publications between 1998 to 2008.**
(XLS)

Table S8 **RNAi-related publications between 1998 to 2008.**
(XLS)
